# Infected Mature Teratoma of Mesentery in a Child

**Published:** 2013-05-15

**Authors:** Mahavir Singh, KN Ratan, Babita Rani, YS Kadian, Sonia Hasija

**Affiliations:** Department of Pediatric Surgery, Pt. BD Sharma PGIMS Rohtak, Haryana, India; Department of Pediatric Surgery, Pt. BD Sharma PGIMS Rohtak, Haryana, India; Department of Community Medicine, Pt. BD Sharma PGIMS Rohtak, Haryana, India; Department of Pediatric Surgery, Pt. BD Sharma PGIMS Rohtak, Haryana, India; Department of Pathology, Pt. BD Sharma PGIMS Rohtak, Haryana, India

**Keywords:** Mature teratoma, Mesentery

## Abstract

Mesenteric teratomas are extremely rare in children. We report a case of 5-year-old girl with abdominal mass and fever. At operation, a multicystic mass with variable consistency found within the leaves of the mesentery of jejunum with pus in it. Histopathology examination showed mature infected teratoma of the mesentery.

## INTRODUCTION

Most common site of teratoma is sacrococcygeal region followed by gonads, mediastinum, central nervous system, retroperitoneum, head and neck etc. [1]. Few case reports on mesenteric teratomas are published [2-6]. Here we report a case of mature infected mesenteric teratoma.

## CASE REPORT

A 5-year-old girl was brought by with the complaint of progressive abdominal distension for last one month. Child was having high grade fever for last one week. On examination the patient was pale. Abdomen was distended and a large well defined, tense mass of 10x8 cm size, slightly mobile in transverse axis of the body, occupying most of the central abdomen found. Her hemoglobin level was 6.5 gm% with microcytic hypochromic anemia and leukocytosis. Other biochemical investigations were within normal limits. X-ray abdomen with chest showed large soft tissue mass in left side of the abdomen with displacement of bowel loops towards the right with calcification in it. Ultrasound abdomen revealed a mixed echogenicity mass (9 cm x 7 cm), mainly composed of cystic areas and calcifications. Computed tomography (CT) revealed a heterogenous soft tissue mass (98 mm x 73 mm) in left lumbar and iliac regions. It showed areas of fat, fluid density, soft tissue attenuation and calcifications; displacing adjacent structures (Fig. 1). Provisional diagnosis was teratoma. Alpha-fetoprotein (AFP) level was normal. At operation, a multicystic mass with variable consistency found within the leaves of the mesentery of jejunum with pus in it (Fig. 2). As mass was inseparable from mesentery so resection of mass with adjacent jejunum was done and gastrointestinal continuity was restored. Post-operative stay was uneventful. Histopathology showed mature epithelial element, cartilage and adipose tissues suggestive of mature teratoma. Patient is on regular follow up and doing well. 

**Figure:**
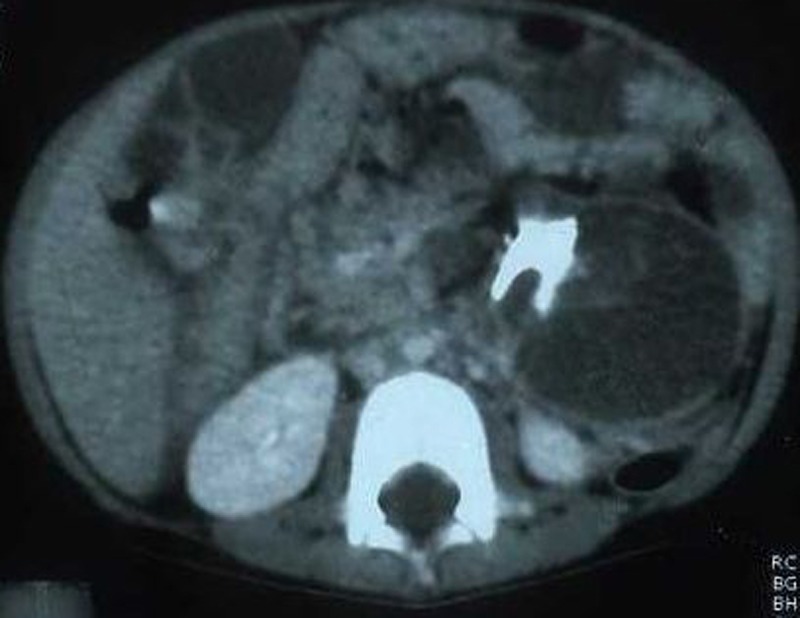
Figure 1: CT scan showing a large heterogenous soft tissue mass showing areas of fat, fluid, soft tissue attenuation with calcifications.

**Figure:**
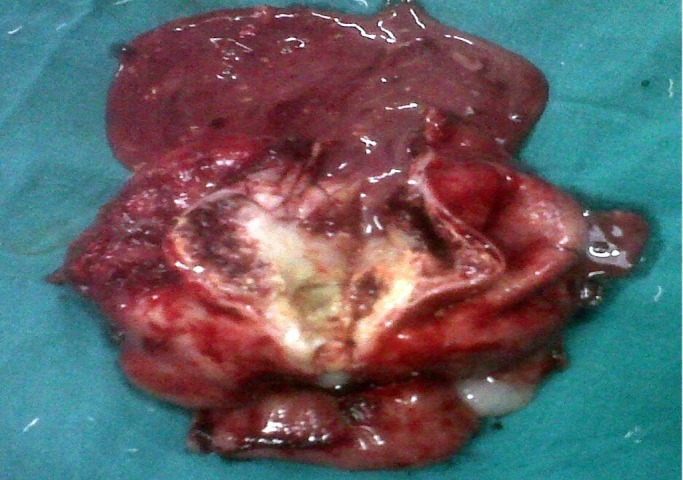
Figure 2: Cut section of excised mass of mesentery along with jejunum showing cystic mass with pus in it.

## DISCUSSION

About 20 cases of mesenteric teratomas have been reported so far; only one was infected [1-6]. Teratomas have no pathognomonic signs or symptoms, and their clinical manifestation depends greatly on the size and location of the growth. The anatomy of the mesentery usually offers sufficient space for considerable growth before symptoms can appear, particularly when the lesion is located near the root. The symptoms may develop early if the lesion is located at the more periphery of the mesentery i.e near the gut lumen. They present frequently, as in our case, with a palpable mass or an increasing abdominal girth. Nausea, vomiting or constipation is the result of intestinal compression by the mass. An intractable chronic diarrhea as a manifestation has also been described [3, 4]. 

Plain abdominal radiograph commonly demonstrates soft tissue mass with calcification noted in nearly 60% of cases. Ultrasound may show an image varying between predominantly cystic to predominantly solid mass with cysts. Also, septations of the cysts can usually be identified. CT is considered more suited for the diagnostic evaluation of gastrointestinal teratomas [5]. Complete surgical excision is the mainstay in the management of intra-abdominal teratoma. Complete tumour resection is sufficient for cure in benign teratoma. Most of the abdominal teratomas are benign in nature and are composed of mature cells. The presence of immature elements in the histology of the excised tumour warrants the need of chemotherapy and regular follow up. Serums AFP assay and ultrasound abdomen are reliable methods of detecting the recurrence; we are also following our case with these modalities [4, 6].

## Footnotes

**Source of Support:** Nil

**Conflict of Interest:** None declared

